# Accounting for Variance in Concussion Tolerance Between Individuals: Comparing Head Accelerations Between Concussed and Physically Matched Control Subjects

**DOI:** 10.1007/s10439-019-02329-7

**Published:** 2019-07-24

**Authors:** Steven Rowson, Eamon T. Campolettano, Stefan M. Duma, Brian Stemper, Alok Shah, Jaroslaw Harezlak, Larry Riggen, Jason P. Mihalik, Kevin M. Guskiewicz, Christopher Giza, Alison Brooks, Kenneth Cameron, Thomas McAllister, Steven P. Broglio, Michael McCrea

**Affiliations:** 1 Department of Biomedical Engineering and Mechanics, Virginia Tech, Blacksburg, VA USA; 2 Department of Neurosurgery, Medical College of Wisconsin, Milwaukee, WI USA; 3 Department of Epidemiology and Biostatistics, Indiana University School of Public Health, Bloomington, IN USA; 4 Department of Exercise and Sport Science, Matthew Gfeller Sport-Related Traumatic Brain Injury Center, University of North Carolina at Chapel Hill, Chapel Hill, NC USA; 5 Departments of Neurosurgery and Pediatrics, UCLA Steve Tisch BrainSPORT Program, David Geffen School of Medicine, University of California, Los Angeles, CA USA; 6 Department of Orthopedics, School of Medicine and Public Health, University of Wisconsin, Madison, WI USA; 7 John A. Feagin Jr. Sports Medicine Fellowship, Keller Army Hospital, United States Military Academy, West Point, NY USA; 8 Department of Psychiatry, Indiana School of Medicine, Indianapolis, IN USA; 9 Michigan Concussion Center, University of Michigan, Ann Arbor, MI USA

**Keywords:** Biomechanics, Impact, Brain injury, Threshold, Football, Sensors

## Abstract

Researchers have been collecting head impact data from instrumented football players to characterize the biomechanics of concussion for the past 15 years, yet the link between biomechanical input and clinical outcome is still not well understood. We have previously shown that even though concussive biomechanics might be unremarkable in large datasets of head impacts, the impacts causing injury are of high magnitude for the concussed individuals relative to their impact history. This finding suggests a need to account for differences in tolerance at the individual level. In this study, we identified control subjects for our concussed subjects who demonstrated traits we believed were correlated to factors thought to affect injury tolerance, including height, mass, age, race, and concussion history. A total of 502 college football players were instrumented with helmet-mounted accelerometer arrays and provided complete baseline assessment data, 44 of which sustained a total of 49 concussion. Biomechanical measures quantifying impact frequency and acceleration magnitude were compared between groups. On average, we found that concussed subjects experienced 93.8 more head impacts (*p* = 0.0031), 10.2 more high magnitude impacts (*p* = 0.0157), and 1.9 × greater risk-weighted exposure (*p* = 0.0175) than their physically matched controls. This finding provides further evidence that head impact data need to be considered at the individual level and that cohort wide assessments may be of little value in the context of concussion.

## Introduction

Researchers have been collecting head impact data from instrumented collegiate football players to characterize the biomechanics of concussion for the past 15 years.^5^,^11^,^12^,^15^,^20^,^24^,^28^,^33^ While these efforts have advanced our understanding of the impact characteristics associated with injury, the link between biomechanical input and clinical outcomes is still not well understood. The biomechanics associated with concussive impacts are often unremarkable when looking at these large datasets as a whole. For any given concussive head acceleration, there might be 1000 other head impacts in the dataset that look just like it and do not cause injury. This leads to the question of: what is unique about the specific impacts causing injury? This paper presents an exploratory analysis aimed at explaining some of the variance in concussion tolerance observed in these datasets.

We have previously published an analysis attempting to relate the presentation of concussion symptoms with biomechanical characteristics of impact.^32^ The underlying rationale was that mechanical input to the head should be related to clinical outcome severity. More specifically, similar biomechanical inputs (impact location and acceleration magnitude) should produce similar injury responses (symptom severity and duration) between injured subjects if all other factors between individuals were equal. A relationship between head impact severity and symptom severity or duration was not observed. Even though concussive impacts did not stand out relative to non-injurious impacts, we did observe that concussive impacts were among the most severe recorded for each subject. We attributed our findings to biological variance resulting in differences in tolerance between individuals. Biological variance, defined here as intrinsic inter-individual differences between human subjects, is commonly observed in injury biomechanics research, and largely explains why a threshold for concussion has not been identified.

There are several intrinsic factors likely contributing to differences in tolerance we see between individuals. We can think of these factors as tolerance modifiers. Head size and shape influence how strains develop in the brain from a given head acceleration.^13^,^26^ A smaller head will produce less strain in the brain than a larger head when experiencing identical head accelerations.^26^ For this reason, tolerance to head acceleration varies between head sizes. Differences in head shape will further add to this variance.^13^ Age is also thought to affect concussion tolerance, particularly when considering differences in brain development between adult and pediatric populations.^7^,^27^ Other tolerance modifying factors are related to differences in material properties of the soft tissue of the brain.^25^ Furthermore, there is evidence that people who have previously sustained a concussion are more likely to experience future concussions than those with no history of concussion.^19^ In other words, a previous injury may additionally influence tolerance. When compounding the variance introduced by these tolerance modifying factors, it should not be surprising that concussive impacts do not seem unique relative to non-injurious impacts experienced by others. However, it may be possible to control for some of these variables at a subject-specific level. It is important to note that extrinsic factors, such as head protection and player position, will also influence concussion risk.

The objective of this study was to control for tolerance modifying factors when assessing the biomechanics of concussion by comparing concussed athletes to physically similar controls. Specifically, we aimed to match concussed subjects with a control subject who had similar physical traits and concussion history. We hypothesized that when accounting for these factors, the concussed subject’s biomechanical impact exposure would be greater than their matched control.

## Materials and Methods

Data included in this study are a subset of the Concussion Assessments, Research, and Education (CARE) Consortium.^4^ Data are specific to the Advanced Research Core of CARE between 2015 and 2017. A total of 510 Division I collegiate football players were recruited to participate from 6 sites: 4 universities and 2 military academies. A central study protocol was approved through the Medical College of Wisconsin’s Institutional Review Board (IRB) and the Human Research Protection Office (HRPO). Each local site’s IRB authorized a reliance agreement with the Medical College of Wisconsin’s IRB before implementing a central protocol. All subjects provided written informed consent before participation.

Subjects that did not report complete relevant demographic information at baseline assessments were excluded from analysis, reducing the cohort to 502 subjects. The cohort consisted of males between the ages of 17 and 23 years (mean ± standard deviation: 19.3 ± 1.30 years). Subjects were between 1.65 and 2.11 m tall (1.87 ± 0.07 m) and weighed between 68.0 and 162 kg (105 ± 18.7 kg). Of these subjects, 223 were white (44.4%), 208 were black (41.4%), 49 were multiple races (9.76%), 13 were Hawaiian Pacific (2.59%), 2 were Indian Alaskan (0.40%), 1 was Asian (0.20%), and 6 were unknown (1.20%). Concussion history varied between subjects: 324 reported 0 previous concussions (64.5%), 141 reported 1 previous concussion (28.1%), 25 reported 2 previous concussions (4.98%), 9 reported 3 previous concussions (1.79%), and 3 reported 4 previous concussions (0.598%). There were 81 subjects (16.1%) who sustained a diagnosed concussion during the study period.

Subjects wore either a Riddell Speed (69.5%), Riddell SpeedFlex (29.9%), or Riddell Revolution (0.6%) helmet (Riddell, Elyria, OH). Helmets were equipped with accelerometer arrays to measure head acceleration for every head impact sustained during games and practices (HIT System, Riddell, Elyria, OH).^1^ The arrays consist of 6 accelerometers that are mounted on an elastomer base to remain in contact with the head throughout impact. Any time a single accelerometer measured greater than or equal to 14.4 g during games and practices, data acquisition were automatically triggered. Data were sampled at 1000 Hz and recorded over 40 ms, which included 8 ms of pre-trigger data. Peak resultant linear acceleration, peak resultant rotational acceleration, and impact location were computed from these data.^9^,^30^ Any recorded event with a peak resultant linear acceleration less than 10 g was excluded from analysis because accelerations under 10 g can be experienced with dynamic movement without head impact.^16^ A total of 424,059 head impacts were recorded during games and practices during the study period. Head acceleration data were captured for 51 concussive events.

There were 49 instances of concussion from 44 subjects that we had complete demographic and biomechanical data. For each of these cases, we aimed to find the concussed subject’s “twin” in the dataset to serve as a matched control. Control subjects were identified as the most physically similar subjects to a concussed subject, regardless of team and position. This approach was taken to account for confounding variables that are thought to influence concussion tolerance. Variables we controlled for were height, mass, race, and number of previous concussions. Subject height and mass were combined into a single variable using body mass index (BMI). BMI was used as an indirect measure of head size, and it is assumed that a subject who is taller and heavier would have a larger head than a subject who is shorter and lighter. With this being a retrospective analysis, a direct measure of head size, like circumference, was not available for analysis. Age was not controlled for because the range of ages at the time of injury (18 to 23 years) was not considered to be on the scale that we would expect to see changes (youth vs. adult vs. elderly).

The process for identifying matched controls for concussed subjects was as follows. Subjects were grouped by race because race is a categorical variable. Numeric variables (BMI and number of previous concussions) had varying scales and needed to be standardized so none was weighted more than another. To standardize, *z*-scores were calculated to center and scale each variable by subtracting the mean and dividing by the standard deviation. Within each race, the Euclidean distances between each concussed subject and all other subjects were calculated based on standardized BMI and concussion history. The subjects with the minimum Euclidean distance to a concussed subject were identified as matched controls.

Biomechanical head impact data were summarized for each concussed subject and matched control. For each concussion case, the period that biomechanical data were summarized over was limited to between the first day of practice and the day of the concussion for both the concussed subject and matched control. Time-limiting was done to control for any effects that the relative timing of head impacts might have on tolerance.^2^,^3^ Head impact exposure measures of frequency and magnitude were computed from these data. The number of head impacts sustained and the number of days with at least one head impact were computed as generalized measures of impact frequency. 95th percentile peak linear head acceleration and 95th percentile peak rotational acceleration were computed as measures of acceleration magnitude for each subject. The number of head impacts greater than or equal to the concussive peak linear acceleration was computed on a matched pair basis as a measure of how frequently high magnitude accelerations were experienced. The concussive peak linear acceleration threshold used to count impacts varied for each pair, dependent on the linear acceleration magnitude associated with concussion. Risk-weighted exposure was also computed, which applies a non-linear transformation to acceleration magnitudes depending on how likely a concussion is to occur based on previously aggregated data and then sums transformed data, as an overall measure that combines magnitude and frequency.^14^,^29^,^35^

The distributions of differences for biomechanical measures between matched pairs were found to be non-normal when using Shapiro–Wilk’s normality test. Therefore one-sided Wilcoxon signed rank tests with a continuity correct were conducted with the alternative hypothesis that the concussed group would exhibit greater biomechanical measures. The mean difference with a 95% confidence interval (CI) was also computed to provide an estimate of effect size.

We hypothesize that physically similar people will have more similar concussion tolerance relative to the variance in the larger cohort, and for this reason, the concussed subject will have experienced greater biomechanical measures over an identical period compared to their physically matched control. However, not every subject had a “twin” in the dataset. In response, we also repeated our analysis looking at only the matched pairs with Euclidean distances less than the median distance (top 50% of matches) to investigate whether our effect sizes increased when comparing biomechanical measures between groups. Effect sizes increasing would provide further evidence that these individual characteristics might influence tolerance.

## Results

### Matched Control Identification

**Figure 1:**
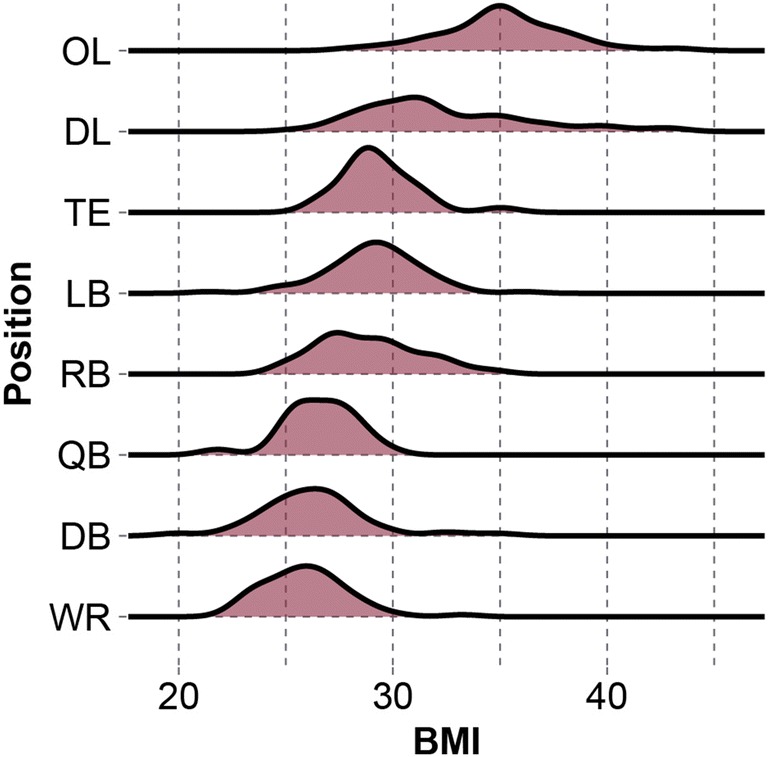
Density plots of BMI distributions across player position groups. Controls were partly identified using BMI and not limited to specific position groups or team.

BMI was used to combine height and weight into a single variable and served as a factor to identify matched controls. Control selection was not constrained to position group, across which BMI varied (Fig. 1).

**Figure 2:**
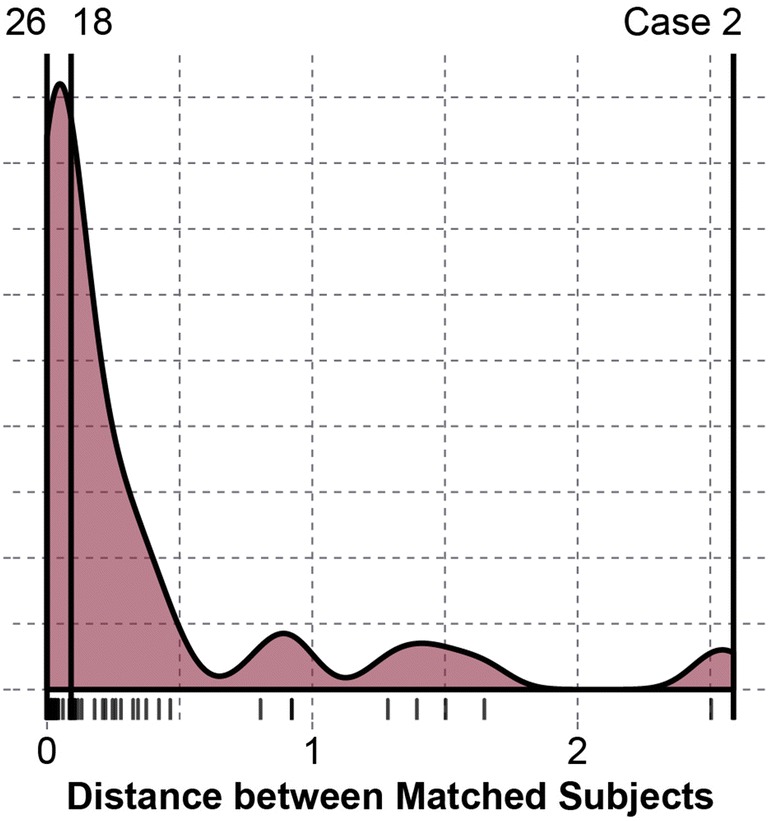
Distribution of Euclidean distances between matched pairs for BMI and concussion history within race. The minimum (Case 26), median (Case 18), and maximum (Case 2) distances are highlighted. Descriptive pairing data are provided in Table 1 for illustrative purposes.

Matched controls were identified for each concussed subject based on minimum Euclidean distance using BMI and concussion history within the race of the concussed subject. The mean distance between matched pairs was 0.370, with a standard deviation of 0.619. The minimum distance between matched pairs was 0.000, and the maximum distance was 2.59. A shorter distance represents a better physical match between subjects. Figure 2 displays the distribution of distances between matched pairs. Table 1 displays exemplary pairing data of varying distances.

**Table 1:** Comparison of race, height, mass, BMI, and concussion history for the matched pairs with the minimum, median, and maximum Euclidean distances.

Case	Distance	Race	Height (m)	Mass (kg)	BMI	Prev. conc.	Position
26	0.000	Black	1.93	113.4	30.4	0	DL
		Black	1.93	113.4	30.4	0	DL
18	0.090	White	1.91	101.2	27.9	1	LB
		White	1.83	94.3	28.2	1	RB
2	2.588	HP	1.96	127.5	33.3	1	OL
		HP	1.88	139.7	39.5	0	DL

Matched pairs were not constrained by position or team. *HP* Hawaiian Pacific, *DL* defensive lineman, *LB* linebacker, *RB* running back, *OL* offensive lineman

### Concussed vs. Match Control Comparisons

**Figure 3:**
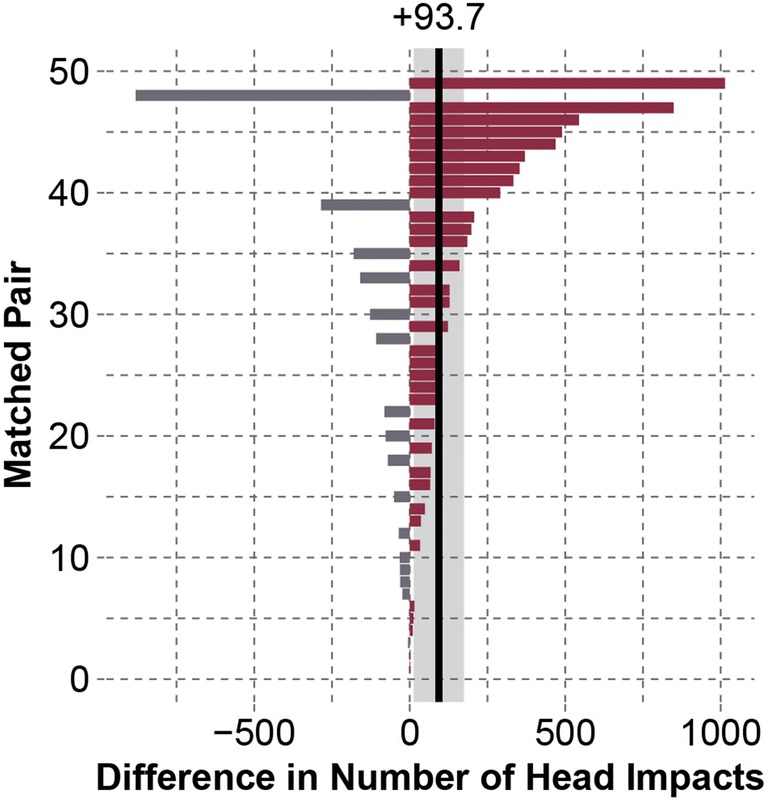
Differences between matched pairs for the number of head impacts sustained over matched periods. A positive value indicates that the concussed player experienced more head impacts. Matched pairs in the plot are numbered in ascending order of absolute difference. On average, concussed players experienced 93.7 [13.4–174] more head impacts than their physically matched controls (*p* = 0.0031).

Concussed subjects, on average, sustained 93.7 [95% CI 13.4–174] more head impacts over matched periods compared to their physically matched controls (*p* = 0.0031) (Fig. 3). During these matched periods, concussed players participated in 4.24 [1.21–7.28] more contact sessions than their controls (*p* = 0.0003).

**Figure 4:**
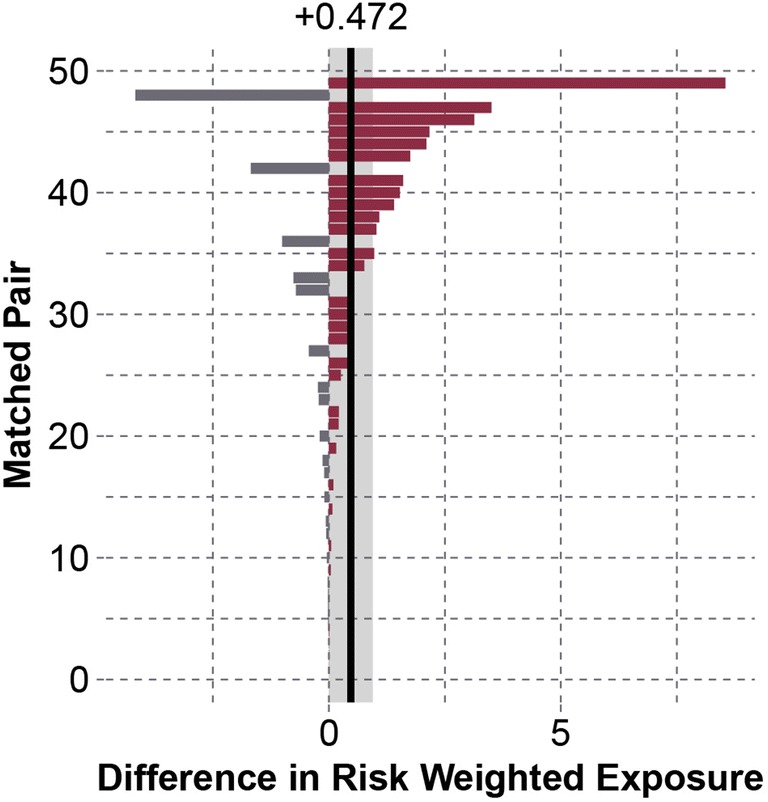
Differences between matched pairs for risk-weighted exposure over matched periods. A positive value indicates greater risk-weighted exposure in the concussed subject. Matched pairs in the plot are numbered in ascending order of absolute difference. On average, concussed subjects experienced 0.472 [0.000 to 0.947] greater risk-weighted exposure than their matched controls (*p* = 0.0175). This represents a 1.90 times greater risk-weighted exposure in the concussed group relative to their physically matched controls.

95th percentile peak linear accelerations in the concussed group were 6.07 g [− 0.48 to 12.6 g] greater than the control group (*p* = 0.033). Concussed subjects also experienced 10.2 [− 1.01 to 21.4] more head impacts associated with peak linear accelerations greater than or equal to their concussive acceleration compared to their matched control (*p* = 0.0157). 95th percentile peak rotational accelerations in the concussed group were 188 rad/s^2^ [− 66.3 to 443 rad/s^2^] greater than the control group (*p* = 0.018). Comparing an overall measure of impact frequency and acceleration magnitude, risk-weighted exposure was 0.472 [0.000 to 0.947] greater in the concussed group (*p* = 0.0175) (Fig. 4). To help provide context to the unit-less risk-weighted exposure values, the mean difference of 0.472 represents a 1.90 times greater risk-weighted exposure in the concussed group than their physically matched controls.

### Comparisons for Best Matches

Not every concussed subject had a physically similar matched control (Fig. 1). To investigate if effect sizes would increase when only comparing good matches, we repeated our analysis on only the matched pairs with Euclidean distances less than the median Euclidean distance. When interpreting the statistics reported below, note that this analysis cut the sample size in half.

**Figure 5:**
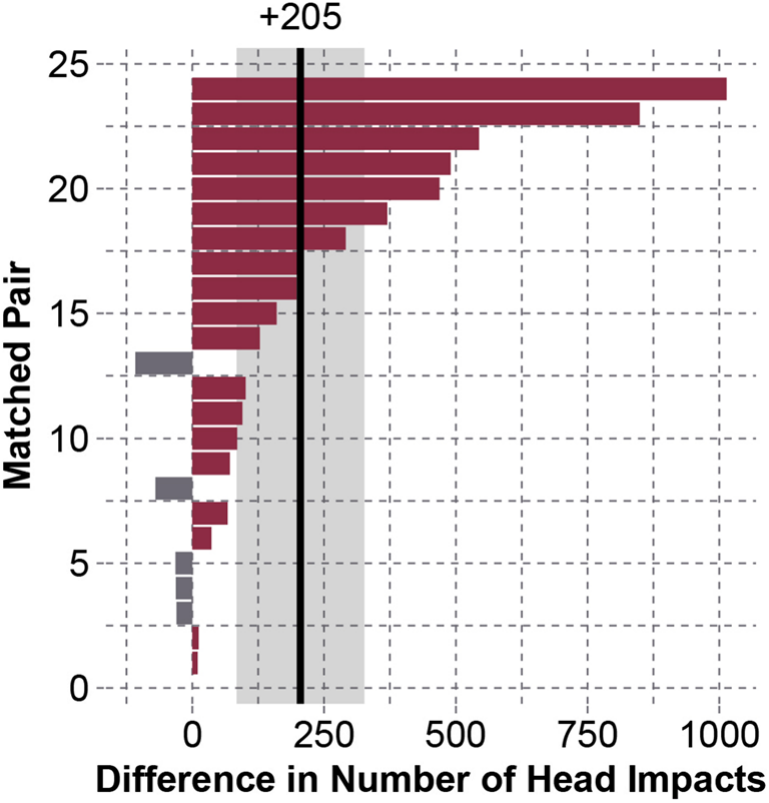
Differences between the 24 most physically similar pairs for the number of head impacts sustained over matched periods. A positive value indicates that the concussed player experienced more head impacts. Matched pairs in the plot are numbered in ascending order of absolute difference. On average, concussed players experienced 205 [84.0–327] more head impacts than their physically matched controls (*p* = 0.0001).

For the best-matched pairs, concussed subjects on average sustained 205 [84.0–327] more head impacts over matched periods compared to their physically matched controls (*p* = 0.0001) (Fig. 5). This difference was associated with concussed players participating in 7.75 [3.04–12.5] more days of contact (*p* = 0.0003).

**Figure 6:**
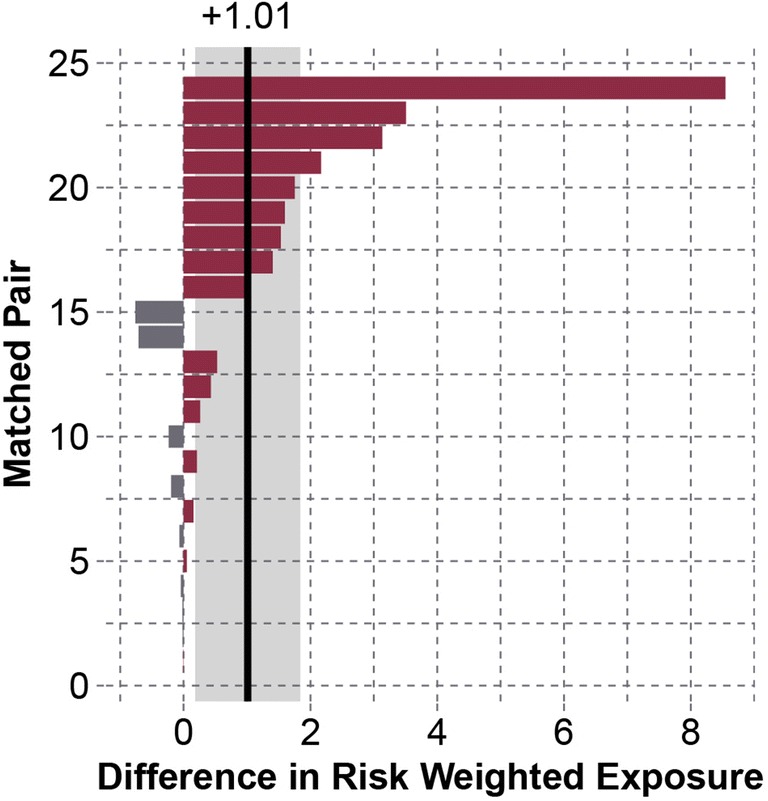
Differences between the 24 most physically similar pairs for risk-weighted exposure over matched periods. A positive value indicates greater risk-weighted exposure in the concussed subject. Matched pairs in the plot are numbered in ascending order of absolute difference. On average, concussed subjects experienced 1.01 [0.184–1.84] greater risk-weighted exposure than their matched controls (*p* = 0.0027). This represents a 3.10 times greater risk-weighted exposure in the concussed group relative to their physically matched controls.

For the best-matched pairs, 95th percentile peak linear accelerations in the concussed group were 7.47 g [− 1.28 to 16.2 g] greater than the control group (*p* = 0.064). Concussed subjects also experienced 17.4 [− 4.31 to 39.1] more head impacts associated with peak linear accelerations greater than or equal to their concussive acceleration compared to their matched control (p = 0.0006). 95th percentile peak rotational accelerations were 202 rad/s^2^ [− 170 to 573 rad/s^2^] greater in the concussed group than control group (*p* = 0.042). Comparing an overall measure of impact frequency and acceleration magnitude, risk-weighted exposure was 1.01 [0.184–1.84] greater in the concussed group (*p* = 0.0053) (Fig. 6). The mean difference of 0.759 represents a 3.10 times greater risk-weighted exposure in the concussed group than their physically matched controls.

## Discussion

We have previously shown that even though concussive biomechanics might be unremarkable in large datasets of head impacts, the impacts causing injury are of high magnitude for the concussed individuals relative to their impact history.^32^ In this study, we identified control subjects who had traits likely to affect injury tolerance that were similar to the concussed subjects. We found that concussed subjects experienced greater impact frequency and acceleration magnitudes than their physically matched controls. It was assumed that each pair minimized differences in tolerance to head acceleration relative to the cohort as a whole. If this is true, the finding that concussed subjects sustained a greater biomechanical load might partially explain why concussed subjects sustained injuries and the controls did not. This finding is not causal but provides further evidence that head impact data collected from athletes wearing sensor systems need to be considered at the individual level and that cohort wide assessments may be of little value in the context of concussion.

Concussed subjects experienced more head impacts than their matched controls leading up to injury. This is not surprising because we also found that concussed subjects participated in over 4 more days of contact than their controls, which is likely an effect of not controlling for player status (i.e., starter vs. non-starter). More cumulative exposure over matched periods may have contributed to instances on concussion.^35^ In addition to elevated impact frequency in concussed subjects, measures of magnitude were also greater in concussed subjects relative to their controls. Concussed subjects experienced more impacts associated with high magnitude accelerations, which are the impacts with the greatest likelihood of producing acute injury from a single impact. We used risk-weighted exposure as a measure to combine impact frequency and acceleration magnitude to summarize overall head impact exposure and found that it was 2 times greater in concussed subjects. While the underlying mechanisms (single impact or repeated impacts) for each injury is not clear, this analysis does suggest the overall biomechanical input can be considered elevated in concussed subjects relative to control subjects exhibiting similar tolerance modifying traits.

Not every concussed subject had a good match in the cohort for the factors we used to identify controls. Poor matches would be considered to have less similar tolerance to head acceleration than good matches, and would not account for the variance in tolerance we attempted to control. For this reason, we performed a secondary analysis that compared concussed subjects to controls for only the best-matched pairs. Interestingly, our effect sizes increased for every measure we evaluated when doing this. However, we cannot say with confidence that this change was meaningful because this analysis also reduced our sample size and widened our confidence intervals. Our non-parametric statistics are likely more telling, where we saw lower *p* values for all measures (even with a reduced sample size) associated with a higher proportion of concussed subjects having greater biomechanical measures than their controls than when considering all pairs. While not conclusive, this provides further evidence that we might be able to control for some variance in concussion tolerance using factors thought to influence tolerance.

Biological variance is common in injury biomechanics research, which has historically been focused on automotive and military applications. The variance we see in the accelerations associated with concussion in our dataset, 73 ± 29 g (coefficient of variation: 43%), is not dissimilar to what is observed for other types of injury. For example, the frontal bone has a fracture force of 1982 ± 765 N (coefficient of variation: 39%).^8^ Furthermore, there is almost always overlap in biomechanical values for injury and non-injury events, meaning there is a range of values where only a proportion of the population gets injured. This overlap is used to model injury risk. In these efforts, the variance in the forces causing injury is built into risk models that are used to predict injury rates for populations. On field head impact measurement in sports is different. Rather than predicting injury rates for athlete populations as a whole, we are primarily interested in predicting injury for specific individuals who are sustaining head impacts. Therefore, our approach to assessing risk should be different than traditional approaches. Our analysis here indicates a need for individual-specific risk analyses that consider a person’s impact history and factors that might influence tolerance to head acceleration.

Other extrinsic factors related to how subjects experience impacts influence risk. Player position will affect head impact exposure measures. For instance, patterns of impact direction within a subject’s impact history will vary by player position.^10^,^11^ Furthermore, we found player position to affect measures of impact frequency more than any other factor, accounting for just under half the variance in frequency after controlling for participation levels in the CARE cohort.^6^ In this study, we did not match control subjects to concussed subjects based on the extrinsic factor of position but rather by the intrinsic factors of height and weight. Figure 1 illustrates how BMI varied by position, and Table 2 shows position pair frequencies used in our analysis. Of matched pairs, 29% played the same position, and 57% played positions that mirrored each other on offense and defense (e.g., offensive lineman and defensive lineman). If a control subject identified to have similar concussion tolerance modifying traits played a position that is less prone to exposure, perhaps that is why the concussed subject sustained an injury and the control did not. Another extrinsic factor affecting risk is head protection.^27^,^31^ Helmets modify the way energy transfers to the head, reducing acceleration magnitude by increasing acceleration duration. While acceleration duration is related to risk, magnitude is thought to be the dominant variable here given that the 3 helmet models used in this study generate durations of similar time domains for matched impacts. For this reason, we did not consider the role of impact duration in our analysis.

**Table 2:** Contingency table describing frequencies of matched pair positions.

Control subjects	Concussed subjects								
	DB	DL	LB	OL	QB	RB	TE	WR	Sum
DB	4	0	4	0	0	0	1	3	12
DL	0	3	0	3	1	1	0	0	8
LB	1	0	2	0	0	0	0	0	3
OL	0	3	1	5	0	1	0	0	10
QB	0	1	1	0	0	0	1	0	3
RB	1	0	1	0	0	0	1	1	4
TE	0	0	0	0	0	1	0	0	1
WR	4	0	1	0	1	2	0	0	8
Sum	10	7	10	8	2	5	3	4	

29% of matched pairs played the same position, and 57% played positions that mirrored each other on offense and defense

*DB* defensive back, *DL* defensive lineman, *LB* linebacker, *OL* offensive lineman, *QB* quarterback, *RB* running back, *TE* = tight end, and *WR* wide receiver

This study has several limitations, and our analysis is based on many assumptions. First, we assumed that the factors we used to identify controls are related to changes in tolerance to head acceleration. Our factors are not exact measures of the traits we aimed to control for but instead served as correlates to the specific traits we believe likely influence tolerance. For example, a relatively heavy and tall subject was assumed to have a larger head than a lighter and shorter subject. Second, the underreporting of concussion is a known phenomenon in football, and it is possible that subjects in the cohort that we used to select controls from had an injury and did not report it.^23^ Given the CARE study protocol and active assessment of potential injury, we believe underreporting was minimized in this dataset.^4^ Concussions were reported in 16.1% of our overall cohort, which is greater than the historical rates of 4.4–5.5% in college football.^21^ Third, we did not consider the effect of impact location and head rotation directionality on brain injury tolerance. It is well known that the brain’s susceptibility to injury varies depending on the plane of rotation, which is a result of the anatomical asymmetry of the brain.^17^,^18^ Future analyses should consider the role of impact location, direction of head rotation, and, if possible, strain-level computations using finite element analysis. Fourth, there are practical challenges to measuring head acceleration data from athletes in the real world and our measurement system is imperfect. The helmet-mounted accelerometer arrays are associated with some measurement error, which can vary in magnitude depending on impact location.^1^,^34^ Fifth, we were unable to measure and analyze rotational velocity, which correlates best to the strain response thought to be associated with concussion.^22^ Sixth, the analysis only compared head impacts for the season in which the concussion occurred for concussed and their matched controls. This approach does not consider head impact history from previous seasons of playing football assumes all subjects baseline status on the first day of practice is the same and not influenced by previous seasons.
